# Icarifil^®^ in Association with Daily Use of Tadalafil (5 mg) versus Standard Tadalafil Daily Dose (5 mg) or Alone: Results from a Controlled, Randomized Clinical Trial

**DOI:** 10.3390/jcm13092564

**Published:** 2024-04-26

**Authors:** Tommaso Cai, Fabrizio Palumbo, Carlos Miacola, Carlo Ceruti, Michele Rizzo, Giovanni Liguori, Luca Gallelli, Alessandro Palmieri

**Affiliations:** 1 Department of Urology, Santa Chiara Regional Hospital, 38122 Trento, Italy; 2 Institute of Clinical Medicine, University of Oslo, 0313 Oslo, Norway; 3 Urology Unit, Di Venere Hospital, 70131 Bari, Italy; palumbo.fab@gmail.com; 4 Department of Urology, University of Bari, 70121 Bari, Italy; carlosmiacola@gmail.com; 5 Department of Urology, University of Turin, 10124 Turin, Italy; 6 Department of Urology, University of Trieste, 34127 Trieste, Italy; mik.rizzo@gmail.com (M.R.); gliguori@units.it (G.L.); 7 Department of Health Science, School of Medicine, University of Catanzaro, 88100 Catanzaro, Italy; gallelli@unicz.it; 8 Department of Urology, University of Naples, Federico II, 80138 Naples, Italy; info@alessandropalmieri.it

**Keywords:** nutraceuticals, erectile dysfunction, *Eruca vesicaria*, *Panax ginseng*, *Tribulus terrestris*, L-arginine, L-citrulline, PDE5-is

## Abstract

**Background**: The management of erectile dysfunction (ED) shows several grey zones and new treatments are required to reduce the percentage of patients discontinuing treatment. Here, we aim to evaluate the role of a natural mixture named Icarifil® (L-Citrulline, L-Carnitine, *Eruca vesicaria*, *Panax ginseng*, *Tribulus terrestris*, *Turnera diffusa*, Taurine, Vitamin E, Zinc) in the management of patients with ED. **Methods**: From September 2022 to March 2023, all patients attending 3 urological institutions due to ED were randomized to receive the following for 3 months: Icarifil® 1 sachet every 24 h (Group 1) or Icarifil® 1 sachet + tadalafil 5 mg 1 tablet every 24 h (Group 2) or tadalafil 5 mg 1 tablet daily (Group 3). All patients underwent urologic visits and dedicated questionnaires (IIEF-5, SEP-2, SEP-3) at enrollment and at the follow-up evaluation (3 months). Patient-Reported Outcomes (PROs) at the follow-up evaluation were used. The primary endpoint was the difference in the questionnaires at the follow-up visit compared to the one at enrollment among the study groups. **Results**: In the per-protocol analysis, 52 patients in Group 1, 55 in Group 2 and 57 in Group 3 were analyzed. At the follow-up evaluation, IIEF-5 scores improved in all the 3 groups between enrollment and the follow-up evaluation, but a statistically significant difference was reported between Group 2 (+7.4) and Group 1 (+4.1) or Group 3 (+5.1), (*p* < 0.001; *p* < 0.001). Moreover, 47 patients (94.0%) in Group 2 showed an improvement in the SEP questionnaires, when compared with the baseline, while 29 in Group 1 (56.9%) and 42 in Group 3 (82.3%) showed a statistically significant difference (*p* = 0.004; *p* = 0.003) among the groups. The PRO analysis reported better efficacy and patient satisfaction in Group 2 when compared with Group 1 or Group 3. **Conclusions**: In conclusion, Icarifil^®^ is able to improve penile erectile function in mild–moderate ED and significantly improve the clinical efficacy of daily used tadalafil 5 mg. Icarifil^®^ could represent an interesting alternative treatment in patients experiencing adverse effects or with contraindications for chronic treatment with PDE5-is.

## 1. Introduction

Erectile dysfunction (ED) is a common pathological condition with severe impact on the quality of life of patients and their partners [1]. Epidemiological studies showed that ED increases with age and is, therefore, much higher in elderly than in young men, but still relatively frequent during middle age [2,3]. The prevalence rates range from 1 to 15% and from 6 to 40% in men aged 30–50 and 50–80 years [4]. In recent years, however, the use of phosphodiesterase-5 inhibitors has increased, especially in young–middle-aged males, demonstrating that the prevalence of ED is rising [5]. Oral phosphodiesterase-5 inhibitors (PDE5-is) represent the first-line pharmacological approach to patients with ED, due to their great efficacy and generally favorable safety profile [6]. However, the percentage of patients who discontinued the treatment is still high. In particular, PDE5-is are associated with several adverse effects, reducing the patients’ compliance and drop-out [7]. On the other hand, several nutraceutical compounds, supplemented as single agents and/or in different combinations, have been reported to offer benefits in the treatment of ED, without adverse effects. Several compounds, such as carnitine, ginseng, *Tribulus terrestris* or damiana, have been reported to show interesting results in terms of clinical efficacy in patients with vasculogenic erectile dysfunction [8,9,10]. Considering the concept that nutraceuticals or phytotherapeutic compounds are considered safer and are generally efficacious for the treatment of mild–moderate ED, some components might represent a valid therapeutic alternative in the treatment of ED alone or in association with PDE5-is. Recently, a mixture of phytotherapeutic compounds named Icarifil^®^, containing L-Citrulline, L-Carnitine, *Eruca vesicaria*, *Panax ginseng*, *Tribulus terrestris*, *Turnera diffusa*, Taurine, Vitamin E, and Zinc has been introduced in the pharmaceutical market for the management of moderate ED. Recently, Amante C. et al. demonstrated, through an in vitro model, that Icarifil^®^ showed efficacy in inhibiting PDE-5 levels higher than 65% compared to the control and that it is able to increase the capability of tadalafil to inhibit PDE5-is, showing a promising possibility of reducing the daily dosage of the drug and consequently its adverse effects [11]. Based on these concerns, we aim to evaluate the role of Icarifil^®^ in the management of patients with ED, by using a randomized and controlled study.

## 2. Materials and Methods

### 2.1. Study Design and Schedule

**Figure 1:**
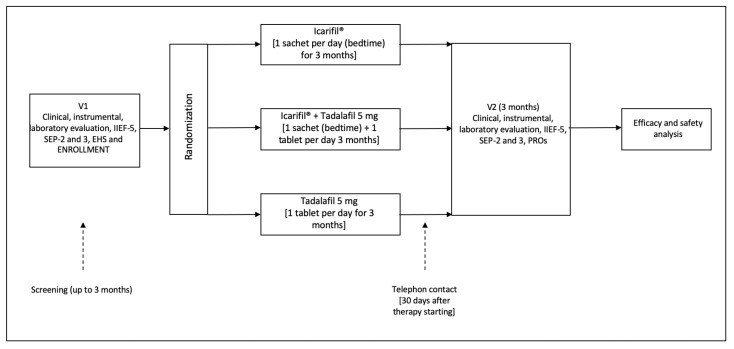
The figure shows the study schedule.

All consecutive patients attending 3 urological institutions due to mild–moderate ED from September 2022 to March 2023 were enrolled in a randomized, controlled phase III study. At enrolment, all patients underwent a urologic visit and completed dedicated questionnaires. Patients were assigned to treatment groups according to a 1:1:1 randomization. Enrolled patients were not blinded. No placebo run-in period was considered necessary. All patients who met the inclusion criteria were randomized by using a computer-generated sequence of allocation as follows: Icarifil^®^ 1 sachet every 24 h (Group 1) or Icarifil^®^ 1 sachet + tadalafil 5 mg 1 tablet every 24 h (Group 2) or tadalafil 5 mg, alone, 1 tablet daily (Group 3). Patients in both groups underwent 3 months of treatment. All patients were contacted by telephone on day 30 of the therapy to the ensure correct timing and dosing of treatment. At the end of the treatment (3 months), all patients underwent a urologic visit and completed dedicated questionnaires. Patient-Reported Outcomes (PROs) at the follow-up evaluation were used. The primary endpoint was the difference among the groups in terms of questionnaire scores between the baseline and end of treatment. Figure 1 shows the study schedule, according to the CONSORT statement [12].

### 2.2. Inclusion and Exclusion Criteria

We enrolled patients aged 18 years or more, affected by mild–moderate erectile dysfunction, according to the results of a 5-question International Index of Erectile Function (IIEF-5) questionnaire [13]. Only patients who scored from 12 to 16 on the IIEF-5 questionnaire were included in the study, according to Tang et al. [14]. Patients with a history of ED on a hormonal basis, secondary to pelvic surgery or purely psychogenic or associated with penile deformity, were excluded.

All patients with major comorbidities were excluded, too. In order to obtain a homogeneous group of patients to analyze, we excluded all patients who had undergone previous treatment with PDE5-is or local alprostadil injections.

### 2.3. Composition and Characterization of the Extracts Used

The intervention schedule included a 3-month treatment with Icarifil^®^ or tadalafil 5 mg or a combination of both. One oral sachet of Icarifil^®^ (registration number 018623914) contained L-Citrulline 1500 mg, L-Carnitine 500 mg, *Eruca vesicaria* (Icariina 5%) 200 mg, *Panax ginseng* (Ginsenosids 80%) 150 mg, *Tribulus terrestris* 100 mg, *Turnera diffusa* (damiana) 100 mg, Taurine 50 mg, Vitamin E 50 mg and Zinc 15 mg, as described in the manufacturer’s instructions (Anvest Health S.p.A, Milan, Italy).

### 2.4. Questionnaires

The validated Italian versions of the Index of Erectile Function (IIEF) [12] and the Sexual Encounter Profile (SEP) questionnaires [15] were filled in by each patient on arrival to the urological outpatient clinics. The questionnaires were collected for each patient at the baseline and at the follow-up evaluation. The IIEF and SEP had been validated in previous clinical trials and had shown good correlation with the response to therapy in patients with ED [16,17]. The erection hardness score (EHS) was used too (3 measurements in 2 separate occasions). The partners of the enrolled patients were also involved in the study by measuring the effectiveness of the therapy using the same questionnaires administered to the patients. Patient-Reported Outcomes (PROs) were also considered in the results analysis, as well as the PROs of the patients’ partners, according to Cai et al. [18].

### 2.5. Statistical Analysis, Outcome Measures and Ethical Considerations

On the basis of the literature data, it is estimated that therapy with Icarifil^®^ can lead to an average improvement in erectile function of approximately 4 points in the IIEF-EF score. On the basis of these considerations, it has been estimated that the study requires 45 patients per group (135 assessments), plus 10% due to the possible drop-out, leading to 150 patients (50 per group). The Chi-square test was used for categorical parameters and changes from baseline to end of therapy were analyzed by ranked one-way analysis of variance (ANOVA). The threshold of statistical significance was set at *p* < 0.05. All reported *p*-values were two-sided. All statistical analyses were performed using SPSS 23.0 (IBM Corporation, Armonk, NY, USA). The primary outcome was to evaluate the change in the erectile function domain score (IIEF-5 and SEP-2 and SEP-3) from baseline to the control visit among the groups. Moreover, PROs’ measure was used. This study was approved by the local ethics committee (approval protocol number 258, 2019) and its was conducted in compliance with the Institutional Review Board/Human Subjects Research Committee requirements and with the Declaration of Helsinki and the Guidelines for Good Clinical Trial Practice criteria. Patients could decide to abandon the study at any time, as specified by the informed consent. An enrolled patient could also be excluded if serious side effects related to the proposed treatment appeared. Randomization was based on a single sequence of random assignments (simple randomization) and performed using a pseudo-random number generator software (Research Randomizer Version 4.0, Social Psychology Network, Wesleyan University, Middletown, CT, USA).

## 3. Results

### 3.1. Patients

From the initial cohort of 177 patients attending our centers in the study enrollment period, 161 met the inclusion criteria and were randomly allocated as follows: 54 to Group 1, 55 to Group 2, and 52 to Group 3. Ten patients were excluded from the final analysis due to missing data at the follow-up evaluation. In the per-protocol analysis, data from 51 patients in the Icarifil^®^ alone group, 50 in the Icarifil^®^ in association with tadalfil 5 mg, and 51 in the tadalafil alone group were gathered. Table 1 shows all demographic, anamnestic, clinical and laboratory data at enrollment.

### 3.2. Follow-Up Results

At the follow-up evaluation, 47 patients (94.0%) in Group 2 showed an improvement in the SEP-2, when compared with the baseline, while 29 in Group 1 (56.9%) and 42 in Group 3 (82.3%) showed a statistically significant difference (*p* = 0.004; *p* = 0.003) among the groups. In the three groups, a statistically significant difference was reported from the baseline to the follow-up in terms of SEP-2 and 3 questionnaires (*p* = 0.002; *p* = 0.003; *p* = 0.003). Moreover, IIEF-5 was improved in all the 3 groups between enrollment and the follow-up evaluation, but a statistically significant difference was reported between Group 2 (+7.4) and Group 1 (+4.1) or Group 3 (+5.1), (*p* < 0.001; *p* < 0.001). The PRO analysis reported a better efficacy and patient satisfaction in Group 2 when compared with Groups 1 or 3. Table 2 shows the follow-up results according to the groups.

### 3.3. Patients and Their Partners’ Reported Outcomes (PROs)

All patients, as well as their partners, were asked about the efficacy of the drugs by comparing the pre- and post-treatment erectile function. All patients in the three groups reported a significant improvement in their erectile function. In the group treated with Icarifil^®^, the reported efficacy seemed better than in the other groups, according to an evaluation using PROs. Their partners confirmed these findings. Moreover, also in the group treated with Icarifil^®^ alone, the daily administration showed a more “natural” treatment strategy because it avoided on-demand administration, even if the IIEF-5 results were lower than in the combination group. Moreover, in all three groups, patients reported an increase in the frequency of spontaneous nocturnal penile tumescence: +47% in Group 1, +79% in Group 2, and +56% in Group 3.

### 3.4. Adverse Effects

Patients treated with Icarifil^®^ alone reported a lower prevalence of adverse effects in comparison with the use of Icarifil^®^ in association with tadalafil or tadalafil alone. In the association group and tadalafil alone group, 7 and 8 patients reported mild adverse effects, respectively. These adverse effects did not require the discontinuation of treatment (lower back pain).

## 4. Discussion

### 4.1. Major Finding

Here, we demonstrated that Icarifil^®^ seems to be an interesting therapeutic approach for patients affected by mild–moderate erectile dysfunction and for all patients in whom an improvement in the clinical efficacy of daily used tadalafil 5 mg was needed. Moreover, the use of Icarifil^®^ seemed to also represent an interesting alternative treatment for patients experiencing adverse effects or with contraindications for chronic treatment with PDE5-is.

### 4.2. Results in Comparison with Other Studies

Several phytotherapy compounds have recently been introduced in everyday clinical practice for managing patients affected by ED. The interest among researchers and clinicians in phytotherapy and nutraceuticals in the management of ED has been increasing in recent years [19]. This is due to the high cost of PDE5-is, the lack of responsiveness in patients with certain comorbidities, and related adverse events of PDE5-is [20]. In this scenario, a new natural mixture, Icarifil^®^, has been introduced in the pharmacological market for modulating the to the nitric oxide/cyclic guanosine 3′5′-monophosphate (NO-cGMP) system and improving erectile function. Amante C. et al. demonstrated that this compound is able to inhibit PDE5 levels higher than 65% compared to the control and 35% compared to a control mixture of L-Citrulline and L-Carnitine [11]. The clinical efficacy of Icarifil^®^ is due to the specific pharmacological properties of each singular component [11]. L-Citrulline is metabolized and conversed in Arginine, which represents a precursor of nitric oxide [21,22]. The choice to use L-Citrulline rather than Arginine is due to the fact that Arginine shows reduced bioavailability due to a significant intestinal pre-systemic metabolization by arginases [21]. On the other hand, L-Citrulline is not metabolized by arginases [22]. Through the same NO/cGMP pathway, *Panax ginseng* is able to induce the vasodilatation of the corpus cavernosum and improve the erectile function [23]. Erectile dysfunction is associated with aging through the penile fibrosis and decreasing smooth muscle and endothelial integrity. The preservation of endothelial function is essential in order to reduce the risk of ED. Icariin and erucine, derivates from *Eruca vesicaria*, are able to preserve penile hemodynamics, smooth muscle, endothelial integrity, and the neuronal expression of nitric oxide synthetases [24], and cause myorelaxation and vasodilatory activity of the smooth muscles [25]. Moreover, Icariin and erucine are able to preserve the inactivation of nitric oxide by oxidative stress [25]. Finally, protodioscin, extracted from *Tribulus terrestris*, is a steroidal saponin precursor of androgens that has also been involved in the nitric oxide synthase pathway determining the relaxation of the corpus cavernosum [26]. It may be the reason for the increased frequency of spontaneous nocturnal penile tumescence in patients treated with Icarifil^®^. It has been previously described that the frequency of decreased nocturnal penile tumescence is one of the most common symptoms highly suggestive of hypogonadism [27]. In this sense, the recovery of spontaneous nocturnal penile tumescence during treatment with Icarifil^®^ is an indirect demonstration of the role of *Tribulus terrestris* in improving the level of androgens. The pre-clinical and clinical efficacy of Icarifil^®^ is due to three different mechanisms of action: one through the androgen pathway, one through the nitric oxide synthase pathway, and one through the anti-ROS and endothelial-and-smooth-cell-integrity-preservation pathway.

Recently, Mirone V et al. reported the results of a clinical study evaluating the role of a nutritional combination of Panax ginseng (500 mg), Moringa oleifera (200 mg) and rutin (50 mg) in the management of patients affected by ED [28]. They compared tadalafil 5 mg once daily plus nutritional supplement once daily with tadalafil 5 mg plus a placebo with the same administration schedule for 3 months. By using a two-phase study, the authors concluded that IIEF-5 showed a significant increase in the combination group, highlighting the antioxidant effects of Moringa oleifera, ginseng and rutin; these can enhance endothelial NO and cGMP production [28]. As reported in our study, Mirone V. et al. highlighted that some nutraceutical compounds are able to improve the efficacy of tadalafil by increasing the nitric oxide synthase pathway and preserving the integrity of endothelial and smooth cells. In our study, the higher increase in IIEF-5, SEP, EHS and PRO questionnaires is probably due to the role of *Tribulus terrestris* in enhancing the endothelial response to PDE5-is through the androgens pathway.

### 4.3. Strengths and Limitations of the Present Study

This study shows important aspects that should be considered, such as the study design and the use of a single phytotherapy compound. This study has been planned as a randomized and controlled study, which should be considered a strength of the study. Moreover, we used a single phytotherapy compound that had been previously tested through the use of an in vitro study, with interesting findings. On the other hand, the present study had a few limitations that should be considered, such as the small number of enrolled patients, even if useful for the statistical analysis and the non-blinded nature of the study. The lack of a wash-out period and the lack of a placebo group should be considered as limitations, too. In order to reduce the impact of the lack of the wash-out period, we excluded all patients who had undergone previous treatment with PDE5-is or local alprostadil injections or other nutraceutical compounds.

## 5. Conclusions

In conclusion, the use of the combination of L-Citrulline, L-Carnitine, *Eruca vesicaria*, *Panax ginseng*, *Tribulus terrestris*, *Turnera diffusa*, Taurine, Vitamin E, Zinc, named Icarifil^®^, seems an interesting therapeutic alternative for patients affected by mild–moderate erectile dysfunction. Moreover, Icarifil^®^ is able to significantly improve the clinical efficacy of daily used tadalafil 5 mg and could be represent an interesting alternative treatment for patients experiencing adverse effects or with contraindications for chronic treatment with PDE5-is. Future research is needed in order to establish the most appropriate treatment schedule and dosage according to the patients’ characteristics.

## Figures and Tables

**Table 1:** The table shows the demographic, anamnestic, clinical and laboratory data of all patients at enrollment. BMI = Body Mass Index; IQR ^†^ = interquartile range.

	Group 1	Group 2	Group 3	
				*p*
Number of enrolled patients	51	50	51	
Age (years)				0.71
Median (IQR ^†^)	51 (42–67)	53 (43–66)	53 (42–69)	
BMI (kg/m^2^)				0.72
Median (IQR ^†^)	28 (26–30)	27 (25–30)	27 (26–31)	
Charlson Comorbidity index (CCI)				0.12
0	49 (96.1)	48 (96.0)	50 (98.1)	
1	2 (3.9)	2 (4.0)	1 (1.9)	
2	0 (-)	0 (-)	0 (-)	
Number of sexual partners				0.37
1	41 (80.3)	39 (76.4)	40 (78.4)	
2 or more	10 (19.7)	11 (23.6)	11 (22.6)	
Sexual partners age				0.84
Median (IQR ^†^)	48 (39–65)	48 (38–66)	49 (39–67)	
Duration of erectile dysfunction (months)				0.91
Median (IQR ^†^)	7 (6–9)	8 (6–9)	8 (6–9)	
Etiology of the disease				0.09
Organic	5 (9.8)	4 (8.0)	5 (9.8)	
Psycogenic	18 (35.3)	20 (40.0)	19 (37.2)	
Mixed	28 (54.9)	26 (52.0)	27 (53.0)	

**Table 2:** The table shows all follow-up results according to the groups in terms of questionnaires. IIEF-5 = Index of Erectile Function (IIEF); SEP = Sexual Encounter Profile; EHS = Erection hardness score; IQR ^†^ = interquartile range; * = difference between Group 2 and Group 1; ^#^ = difference between Group 2 and Group 3.

	Group 1		Group 2		Group 3		
							*p*
	Baseline	Follow-Up	Baseline	Follow-Up	Baseline	Follow-Up	
IIEF-5							<0.001 * 0.82 ^#^
Median (IQR ^†^)	15 (13–15)	19 (18–21)	14 (13–15)	23 (21–25)	14 (13–15)	22 (21–25)	
*p*	<0.003		<0.001		<0.001		
SEP-2							<0.001 * <0.001 ^#^
Positive response (%)	19 (37.2)	29 (56.9)	19 (38.0)	47 (94.0)	20 (39.2)	42 (82.3)	
*p*	<0.001		<0.001		<0.001		
SEP-3							<0.001 * <0.001 ^#^
Positive response (%)	11 (21.5)	30 (58.8)	13 (26.0)	47 (94.0)	12 (23.5)	43 (84.3)	
*p*	<0.001		<0.001		<0.001		
EHS							<0.001 * 0.09 ^#^
Median (IQR ^†^)	2 (1–3)	3 (3)	2 (1–3)	4 (3–4)	2 (1–3)	3 (3–4)	
*p*	<0.001		<0.001		<0.001		

## Data Availability

Data are unavailable due to privacy or ethical restrictions in accordance with Italian bylaw.
